# Resiniferatoxin and Tetrodotoxin Induced NPY and TH Immunoreactivity Changes Within the Paracervical Ganglion Neurons Supplying the Urinary Bladder

**DOI:** 10.1007/s12031-012-9889-z

**Published:** 2012-10-02

**Authors:** Piotr J. Burliński, Anna M. Burlińska, Sławomir Gonkowski, Jarosław Całka

**Affiliations:** Faculty of Veterinary Medicine, Olsztyn, Poland

**Keywords:** NPY, TH, Paracervical ganglion, Immunohistochemistry, RTX, TTX

## Abstract

Both resiniferatoxin (RTX) and tetrodotoxin (TTX) have been reported to be effective in several urinary bladder dysfunction clinical trials. The aim of this study was to establish the effect of intravesical administration of RTX and TTX on neuropeptides Y (NPY) and tyrosine hydroxylase (TH) relationship in the paracervical ganglion (PCG) neurons supplying the urinary bladder in the pig. TH is an enzyme responsible for catalyzing the conversion of the amino acid L-tyrosine to dihydroxyphenylalanine (DOPA) and is used as a marker of catecholaminergic neurons. NPY augments the vasoconstrictor effects of noradrenergic neurons, and is involved in pathophysiological processes as a neuromodulator. To identify the PCG neurons supplying urinary bladder Fast Blue (FB) was injected into the bladder wall prior to intravesical RTX or TTX administration. Consequent application of immunocytochemical methods revealed that in control group 64.08 % of FB-positive PCG neurons contain NPY and 4.25 % TH. Intravesical infusion of RTX resulted upregulation of the NPY-IR neurons to 82.97 % and TH-IR to 43.78 %. Also administration of TTX induced further increase number of TH-IR neurons to 77.49 % but induced decrease number of NPY-IR neurons to 57.45 %. Both neurotoxins affect chemical coding of the PCG neural somata supplying urinary bladder, but the effects of their action are different. This results shed light on possible involvement of RTX and TTX on curing tissue, and potentially could help us to broaden our neurourological armamentarium.

## Introduction

The treatment of micturition disorders, such as overactivity/hyperactivity of the bladder, remains a problem for neuro-urologists. It is suggested that a good tool for the urinary incontinence treatment is neurotoxins, and studies continue on the inclusion of some of them in our neurological resources. In the last few years, for example, O-conotoxin GIVA, botulinum toxin, guanethidine, resiniferatoxin (RTX) and tetrodotoxin (TTX) were intensively studied (Radziszewski and Borkowski 2001; Apostolidis et al. 2006; Bossowska et al. 2008; Bossowska et al. 2009; Lepiarczyk et al. 2010; Lew et al. 2010a; Lew et al. 2010b; Burliński et al. 2011; Burliński et al. 2012). The evaluation of neurotoxin influence on the chemical coding of the neurons supplying the urinary bladder may increase our knowledge about the consequences of their action on treated tissue and organisms. This may help to broaden neuro-urological armamentarium by enabling a better choice of treatment for specific micturition disorders.

Resiniferatoxin is a thousand times more potent analog of capsaicin. Its action is via the vanilloid receptor, affecting the nonmyelinized C fibers of the afferent neurons which causes paralysis of the sensory cells. Intravesical application of RTX evokes desensitization or degeneration of nerve endings of the bladder wall which induces changes in the chemical coding of neurons (Apostolidis et al. 2005). If used as a drug, it can stop the afferent neuron activity and involve in anomalous neural reflexes causing neurogenic micturition disorders. On the other hand, tetrodotoxin exerts its action through the sodium channels of nerve cells. In medicine TTX has been used to treat leprosy, tetanus, rheumatoid arthritis, migraines, and cardiac arrhythmia. It is also applied in allodynia elimination and as a pain reliever in the terminal stage of cancer (Suehiro 1994; Marcil et al. 2006; Shi et al. 2009).

The paracervical ganglion (PCG) is a unique and very interesting structure. It is also one of the sources for innervating the urinary bladder and contains both sympathetic and parasympathetic neurons (Keast 1995a, 1999; Vaughan and Satchell 1995; Podlasz and Wąsowicz 2008). Former reports have confirmed the presence of cholinergic, adrenergic, and nonadrenergic-noncholinergic neurotransmitters, as well as neuropeptide Y in the pelvic ganglia including female PCG and its male counterpart in laboratory animals and pig (Papka et al. 1987; Mitchell 1993; Gibbins 1995; Keast 1995b; Keast et al. 1995; Podlasz and Wąsowicz 2008). Tyrosine hydroxylase (TH) is used as a marker of catecholaminergic neurons, while neuropeptides Y (NPY), as well as ChAT/VAChT, VIP, and SOM, is the most frequently observed substances in PCG neurons, which testifies its important function in this nerve's structure (Podlasz and Wąsowicz 2008; Bossowska et al. 2009).

Pigs show embryological, anatomical, and physiological similarities to humans. For this reason it is an especially valuable species for biomedical research, including lower urinary tract disorders (Crowe and Burnstock 1989; Swindle 1992; McMurray et al. 2006; Verma et al. 2011).

There is a deficiency of data concerning the effect of RTX and TTX on the chemical plasticity of bladder-supplying PCG neurons. The purpose of this study was therefore to determine the co-localization of NPY and TH in neurons of the PCG innervating the urinary bladder in pig after intravesical RTX and TTX application.

## Materials and Methods

For the purpose of this experiment, nine juvenile female pigs of the Large White Polish breed were used (12–18 kg of body weight), which are obtained from a farm in Bałcyny (Poland). The animals were kept in standard laboratory conditions with admission to species and age-specific chow and water ad libitum. They were equally divided into one control and two experimental groups. All surgical procedures were performed in compliance with the instructions of the national ethical committee, with special attention paid to the minimizing of pain and any stress reaction, following the decision of the Local Ethical Committee in Olsztyn, number 35/N and dated 11 June 2005.

In order to identify the PCG neurons that innervate the bladder, laparotomy was performed under sodium thiopental anesthesia (Thiopental, Sandoz, Kundl-Rakúsko, Austria; 20 mg/kg of body weight given intravenously), and a total of 80 μl of 5 % Fast Blue (FB) suspension (EMS-Chemie, Germany) was evenly injected into the left, right, and ventral wall of the urinary bladder in portions of 2 μl each. The FB suspension was injected by using a Hamilton microsyringe equipped with a 26-gauge needle.

After two weeks the animals from the experimental groups were treated with intravesical RTX (LC Laboratories) (experimental group 1) and intravesical TTX (Alexis Biochemicals) (experimental group 2) infusion using an appropriate-sized catheter. Doses of 300 μg of RTX in 60 ml of 5 % ethanol per animal and 12 μg of TTX in 60 ml citrate buffer (pH 4.9) per animal were applied. Ten minutes after the infusion, the contents of the bladder were evacuated and the catheter was removed. The aqueous insolubility of RTX necessitates the use of ethanol as a solvent for the instillation of this drug. In clinical trials 30 or 10 % ethanol which was prepared as the same on humans was used as a vehicle solution (Silva et al. 2000; Kuo 2003; Shin et al. 2006). Thus, the ethanol mixture that contained RTX was used in this experiment. The high concentration of alcohol may evoke vesical pain or temporarily misalign the work of the urinary tract in juvenile pigs. For these reasons only 5 % ethanol solution were used.

Three weeks following FB injection, animals from all three groups were euthanized by an overdose of sodium thiopental prepared ex tempore and then perfused transcardially with 4 % paraformaldehyde in 0.1 M phosphate buffer (pH 7.4). The uteri were then exposed, and the uterine cervices with paracervical ganglia were collected. The tissues were post-fixed for 30 min in the same fixative, rinsed in buffer, transferred to 18 % sucrose in 0.1 M phosphate buffer (pH 7.4), and stored at 4 °C for two weeks.

Ten-micrometer-thick cryostat transverse sections were processed for double-labeling immunofluorescence as described previously by Pidsudko et al. (2001), using combinations of immunosera raised in different species and directed towards rabbit anti-NPY (cat. nr NA 1233, Biomol, 1:4,000), and mouse anti-TH (cat. nr MAB 318, Chemicon Millipore, 1:100). The primary antisera were visualized by species-specific secondary antisera conjugated to FITC or biotin (all from Jackson Immunochemicals, USA, in a working dilution of 1:800). The latter antibodies were then visualized by a streptavidin–CY3 complex (Jackson, 1:8,000). The negative controls employed in the immunofluorescence procedure included preabsorption of the neuropeptides with an appropriate antigen, where the primary antibodies were omitted from the applied staining protocol. For the replacement control, the antibodies were replaced by a normal rabbit or mouse serum at a respective dilution. Staining was not observed in either case.

At the beginning, the PCG-located FB-positive neurons were identified under a fluorescent microscope and then the nerve cells were examined for NPY and TH immunoreactivity. To avoid counting the same neurons twice, the traced neuronal somata were counted in every fourth section.

Statistical data obtained from each of the investigated groups were subjected to a comparative analysis to determine the quantitative variability of the presence/absence of TH and NPY in FB-positive neurons. The significance of differences was estimated using ANOVA and Duncan’s tests. The highly significant differences (*p* ≤ 0.01) in accordance with the generally prevailing principles are indicated in the Table 1 in single asterisk, while the significant differences (*p* ≤ 0.05) are indicated using double asterisk. Data were processed statistically using the STATISTICA 9.0 application (StatSoft Inc., Tulsa, Oklahoma, USA).

**Table 1:** Average percentage of FB-IR single immunostaining neurons containing the studied substances tested in different experimental groups

		FB+		Number of neurons
		NPY-IR	TH-IR	
Control group	Average (%)	64.08*^,^**	4.25*	1,258
	SEM	1.44	3.67	
RTX	Average (%)	82.97*	43.78*	1,300
	SEM	2.62	1.19	
TTX	Average (%)	57.45**	77.49*	1,192
	SEM	3.81	1.55	

Data are shown as the averages ± standard error of means (SEM) for the three data points per group. The significance of differences was estimated using the Duncan's test. The statistical significance was evaluated between the neuronal group presenting the same neurochemical characteristics

(*p ≤ 0.01; **p ≤ 0.05)

## Results

The most numerous concentrations of bladder-innervating PCG-retrogradely labeled neurons were identified in the utero-vaginal transition zone. The neuronal population gradually diminished in both cranial and caudal directions.

**Fig. 1:**
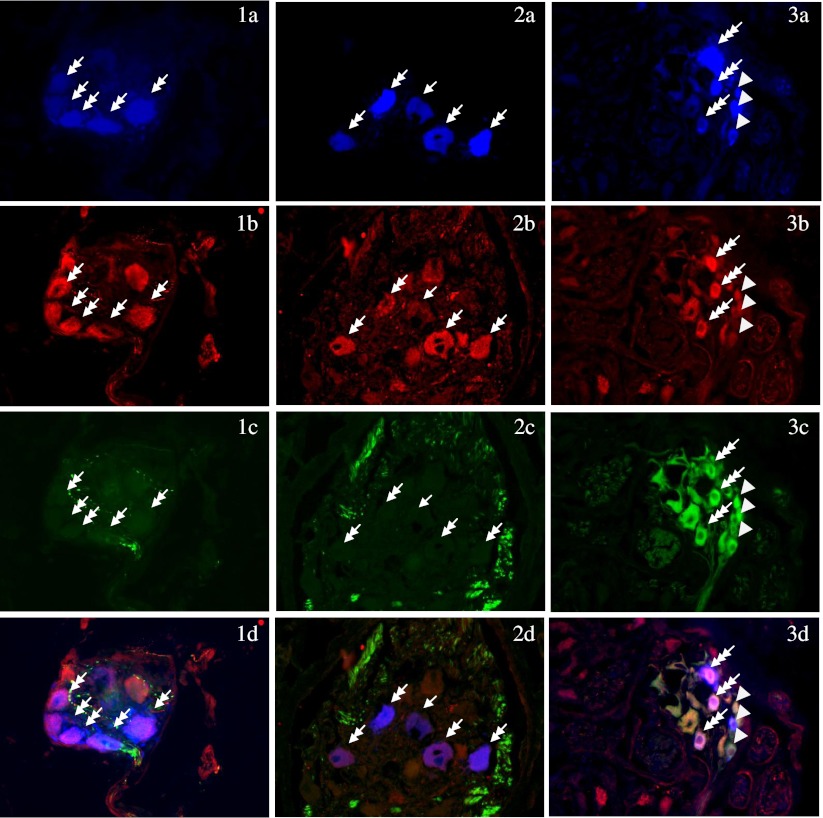
Porcine paracervical ganglion sections from control group (*1*) and after RTX (*2*) and TTX (*3*) instillation, triple-labeled for Fast Blue (*a*), NPY (*b*), and TH (*c*), and combined image (*d*) (magnification in *1*, *2*, ×200 and *3*, ×100). *Single arrows* indicate retrogradely traced, exclusively FB-positive PCG perikarya, while *double arrows* point out double-labeled FB+/NPY+/TH− cell bodies. *Triple arrows* show triple-labeled FB+/NPY+/TH + neurons; *triangle* indicates FB+/NPY−/TH + nerve somata

Double immunocytochemical staining confirmed that in the control group, 64.08 % of the FB-positive neurons simultaneously expressed NPY immunoreactivity, while only 4.25 % were tyrosine hydroxylase-immunoreactive (TH-IR). Additionally, FB+/NPY−/TH+ neurons represented 2.34 % of the total population of FB-positive cells. Triple-labeled FB+/NPY+/TH+ somata accounted for 1.91 % of the FB-positive neurons. The number of neurocytes FB+/NPY+/TH− was 62.17 %, while 33.58 % of the FB-labeled neurocytes were lacking both NPY- and TH-IR (Fig. 1 1a–d).

In the RTX treated group, 82.97 % of the FB-positive neurons simultaneously expressed NPY and 43.78 % expressed TH immunoreactivity. The FB+/NPY−/TH+ neurons represented 4.18 % of the FB-positive cells, while FB+/NPY+/TH+ neurons constituted 39.6 % of the FB-positive cell bodies. Of the PCG neurons innervating the urinary bladder, 12.84 % were lacking both NPY- and TH-IR (Fig. 1 2a–d).

In the group treated with intravesical instillation of TTX, the population of cells containing both FB and NPY was 57.45 %, while 77.49 % of FB+ cells also expressed TH. The FB+/NPY−/TH+ neurons represented 30.19 % the FB-positive cells, while 12.35 % out of the total FB-positive population were negative for NPY and TH (Fig. 1 3a–d) (Tables 1 and 2).

**Table 2:** Average percentage of neurons containing the studied substances tested in different experimental groups

		FB+			
		NPY+/TH+	NPY+/TH-	NPY−/TH+	NPY−/TH−
Control group	Average (%)	1.91*	62.17*	2.34*	33.58*
	SEM	1.65	2.63	2.02	1.86
RTX	Average (%)	39.6*	43.37*	4.18*	12.84*
	SEM	1.69	1.54	1.02	2.61
TTX	Average (%)	47.3*	10.15*	30.19*	12.35*
	SEM	3.23	0.60	1.69	2.13

Data are shown as the averages ± SEM for the three data points per group. The significance of differences was estimated using the Duncan's test. The statistical significance was evaluated between the neuronal group presenting the same neurochemical characteristics

(*p ≤ 0.01)

Statistical analysis revealed highly significant differences (*p* ≤ 0.01) in quantities of NPY- and TH-positive neurons between the groups. Only between the control and TTX group that the significant differences in the number of neuropeptides Y-immunoreactive (NPY-IR) neurons (*p* ≤ 0.05) (Table 1) were seen.

Highly significant differences (*p* ≤ 0.01) in the percentage average were found between all groups for NPY+/TH+ and NPY+/TH− neurons. There were neither statistical differences between the control and RTX group for only TH-IR neurocytes nor between RTX and TTX group for only FB-positive neural somata. In other cases, the differences were statistically highly significant (*p* ≤ 0.01) (Table 2).

## Discussion

The present investigation reports on the effect of an intravesical administration of RTX and TTX on NPY and TH expression in paracervical ganglion neurons supplying the urinary bladder in the pig. The accepted vehicle for intravesical capsaicin is 10 or 30 % ethanol. Unfortunately, in such concentration the ethanol vehicle alone was noted to be irritating to the bladder mucosa. On the other hand, ethanol potentiated the response of vanilloid receptor-1 to capsaicin and their analogs (de Sèze et al. 1998; Trevisani et al. 2002; Kuo 2003; Shin et al. 2006). After using 10 % ethanol, a small number of inflammatory cells in the lamina propria were reported (Silva et al. 2001). To minimize the inflammation effect, RTX was applied in 5 % ethanol.

A single dose of high-concentrated RTX was reported effective, thus this method of treatment was chosen (Kuo 2003; Lazzeri et al. 2004; Watanabe et al. 2004). Silva et al. (2001) reported no histopathological differences between the samples of bladder biopsies obtained from the human patients treated with 50 or 100 nmol/L resiniferatoxin dissolved in 10 % ethanol. For this reason the higher concentration of RTX was used in experiment.

NPY is one of the most important peptidergic transmitters in both the sympathetic and parasympathetic nervous system (O'Donohoue et al. 1985; Lindh et al. 1989; Klimaschewski et al. 1996). Podlasz and Wąsowicz (2008) reported that 75 % of all PCG neurons were NPY-IR although 23 % were TH-IR. Our data indicate that about 64 % of FB-positive neurons were simultaneously NPY-IR while less than 5 % were TH-IR. Both these neurotoxins induced an increase in the TH immunoreactivity in the population studied. Interestingly, RTX provoked an almost 20 % increase in the amount of NPY immunoreactivity, whereas TTX induced a decrease. Podlasz and Wąsowicz (2008) established that most of TH-IR neurons were located in the cranial part of the PCG and supplied by the hypogastric nerve. In our study most of TH-IR neurons, after neurotoxin treatment, were observed more towards the caudal part. TH-IR neurons were quite rare, which is consistent with the previous observations of the pelvic ganglia (Kepper and Keast 2000). These data confirmed that most NPY-IR neurons were not noradrenergic but cholinergic.

Zoubek et al. (1993) have demonstrated that NPY-inhibited nerve stimulation induced contractions. Also, Lundberg et al. (1984) established that NPY induced a reversible reduction of the noncholinergic, nonadrenergic contractile response to field stimulation of the urinary bladder. Electrophysiological studies have shown that neuropeptide Y acts as a modulator of voltage-activated Ca^2+^ channels, which play important roles in synaptic transmission and neuronal excitability (Zhu et al. 1995; Zhu and Yakel 1997). It is also known that TTX, used in medicine as a pain reliever and in allodynia elimination, acts on the sodium channels of the nerve cells (Marcil et al. 2006; Shi et al. 2009). It is interesting that only about 6 % of NPY-IR neurons reacted in response to tetrodotoxin treatment.

Tran et al. (1994) reported that in rat urinary tract NPY elicits two opposed responses, an indirect disinhibitory action to eliminate heterosynaptic cholinergic inhibition of norepinephrine release and a direct inhibitory action on adrenergic nerve terminals. The latter effect correlates well with the previous results regarding the dramatic reduction of ChAT-immunoreactivity in PCG neurons supplying the urinary bladder after TTX treatment (Burliński et al. 2011). The upregulation of LENK and vasoactive intestinal polypeptide (VIP) immunoreactivity evoked by both studied neurotoxins (Burliński et al. 2012) should also be noted. These data suggest that TTX exerts a stronger relaxant effect than RTX and may be more effective in overactivity/hyperactivity bladder treatment.

Our results suggest an important role for NPY- and TH-IR neurons in the regulation of micturition processes. Zhu et al. (1995) indicate a low level of NPY-IR structure in women with pelvic organ prolapse and stress urinary incontinence, while our results indicate that RTX evoked a significant increase in the number of NPY-IR structure. That occurrence is desirable and may indicate the better usefulness of RTX than TTX in this type of low urinary tract disorders. However, the exact biological response of the organism to neurotoxin treatment, especially their damaging or toxic side effects, remains to be explored.
